# Menstrual Cycle Characteristics and Perceived Impact in Female Volleyball Players

**DOI:** 10.3390/sports14060241

**Published:** 2026-06-11

**Authors:** Zsuzsanna Kneffel, Tímea Kováts, Anna Áder, Bence Kopper

**Affiliations:** 1 Department of Health Sciences and Sport Medicine, Hungarian University of Sports Science, 1123 Budapest, Hungary; kneffel.zsuzsanna@tf.hu (Z.K.); timea.kovats@gmail.com (T.K.); ader.panka.mozgas@gmail.com (A.Á.); 2 Department of Kinesiology, Hungarian University of Sports Science, 1123 Budapest, Hungary

**Keywords:** menstrual phases, performance level, volleyball players

## Abstract

Background: Research on the influence of the menstrual cycle on female athletic performance remains limited. This study investigated menstrual cycle characteristics, menstrual disorders, and phase-specific variations in perceived performance among female volleyball players. Methods: Eighty-four recreational and competitive athletes (M = 25.62 ± 6.43 years) completed a comprehensive survey between March and April 2025, including a modified Menstrual Distress Questionnaire (MEDI-Q) assessing physical and psychological well-being, perceived sport performance, training quality, and motivation across the menstrual, follicular, and luteal phases. Results: Perceived sport performance differed significantly across phases, with the highest scores in the follicular phase (M = 1.70 ± 1.51), followed by the luteal (M = 0.88 ± 1.13) and menstrual (M = 0.64 ± 1.00) phases (*p* < 0.001). Perceived performance impairments were greatest during menstruation and lowest in the follicular phase. Motivation exhibited a similar trend, peaking in the follicular (M = 1.74 ± 1.55) and declining during menstruation. Menstrual disorders were reported by 75% of participants, and 59.5% experienced dysmenorrhea. Knowledge scores (M = 11.13/18) indicated a moderate understanding of menstrual physiology. Conclusions: These findings demonstrate significant menstrual phase-related variations in subjective performance and motivation, emphasizing the importance of menstrual cycle awareness, athlete education, and individualized, phase-based training strategies to optimize performance and support female athlete welfare.

## 1. Introduction

The proportion of female athletes at the Olympic Games has risen substantially in recent decades, reaching full gender parity in Paris 2024. Despite this progress, research dedicated to understanding the impact of the menstrual cycle on elite female athletic performance remains comparatively sparse. Although women now comprise approximately half of the elite athletic population, the consideration of cyclic hormonal fluctuations—including estrogen, progesterone, luteinizing hormone (LH), and follicle-stimulating hormone (FSH)—remains underrepresented in sports science research, despite well-documented effects on physiological function, fertility, and injury risk [1,2,3,4,5]. In addition to these objective physiological changes, variations in subjective mental and physical well-being across menstrual cycle phases may also affect performance [1].

The menstrual cycle, driven by pituitary and ovarian hormones, is divided into two principal phases: follicular and luteal. The cycle commences with menstruation (day one), followed by rising estrogen levels throughout the follicular phase, peaking with ovulation. The subsequent luteal phase is characterized by increased progesterone and a secondary elevation in estrogen. Both FSH and LH peak at ovulation. However, there is no universal agreement on the precise subdivision or naming of phases; some frameworks split the follicular and luteal stages further into early, mid, and late, complicating comparisons across studies [3,4,5]. High-intensity physical activity has been correlated with more frequent menstrual irregularities and amenorrhea among females.

High-intensity physical activity has been correlated with more frequent menstrual irregularities and amenorrhea among female athletes [1]. While daily hormone measurements are required to detect subtle disruptions such as anovulation or luteal phase defects, more severe conditions (amenorrhea, oligomenorrhea) should also be diagnosed clinically [6,7,8]. Dusek (2001) [9] identified that secondary amenorrhea occurred three times more frequently in athletes than in non-athletes, while dysmenorrhea was less common among athletes. Similarly, the timing of menarche is delayed in those who start training prior to its onset. Mountjoy et al. (2018) [10] highlighted that elite female athletes are particularly affected by a range of gynecological issues, including menstrual irregularities, delayed menarche, reduced fertility, and urinary incontinence, which often go unreported due to stigma or fears of impairment to training or competition prospects. There is further evidence linking the menstrual cycle to variation in physical and mental aspects of performance, injury risk, and psychological state [1].

Experimental studies have yielded mixed results regarding the phase-specific influence on endurance, strength, and skill-based performance: some studies observed best physiological performance during menstruation, while others documented endurance decrements during the luteal phase [11,12].

Survey-based studies provide insights into athletes’ subjective experiences. For example, Kishali et al. (2006) [13] reported that menstrual disorders were present in 14.5% of athletes during regular training and increased to 20.7% during periods of intense training. They found that 36.9% of athletes experienced menstrual pain, 45.6% reported occasional pain, and 63.1% noted reduced pain during competitions. Training and competition therefore appeared to alleviate pain for many athletes, and non-pharmacological strategies were used more frequently than medication, particularly during competitive periods. Regarding subjective experience, 71% of athletes felt worst immediately before menstruation, yet 62.2% perceived no change in performance during menstruation itself, and only 21.2% reported a decline in performance compared with other phases. In one survey of elite athletes, 76.8% reported their cycle negatively affected performance, highlighting the late luteal (40%) and early follicular (35%) phases as the most detrimental [1]. However, findings remain inconclusive, highlighting the need for further research, particularly regarding subjective performance outcomes.

Knowledge about menstrual cycles among athletes and coaches remains limited, contributing to widespread misconceptions and persistent stigma. Laske et al. (2024) reported that both athletes and coaches frequently cite insufficient knowledge as a key reason for poor communication about the menstrual cycle, highlighting persistent taboos in sports environments [11]. Others have also demonstrated substantial gaps in menstrual-cycle knowledge among both athletes and coaches and emphasized that coaches often feel underprepared to support athletes, which is particularly concerning for volleyball players who must compete across all cycle phases and may therefore be disproportionately affected by insufficient awareness and phase-specific guidance [12]. Together, these findings reveal a clear gap between inconclusive objective performance data and consistent subjective reports of menstrual-cycle-related performance changes in athletes. The present study’s primary outcomes were subjective performance and training quality, while menstrual cycle knowledge among adult volleyball players was assessed as a secondary outcome across menstrual phases (menstruation, follicular, luteal).

## 2. Materials and Methods

### 2.1. Participants

Overall, 84 female recreational and competitive volleyball players were recruited for the study between 30 March and 16 April 2025. Participant characteristics included: mean age of 25.62 (SD = 6.43) years, mean menarche age of 12.73 (SD = 1.34), and 11.90% with a history of childbirth. Exclusion criteria were the following: (a) pregnancy, (b) breastfeeding, or (c) postmenopausal status at the time of data collection. Participants had an average of 12.65 years (SD = 6.33) of volleyball experience. Twenty-six individuals (30.95%) were non-registered players (recreational players), and 58 individuals (69.05%) were registered players competing in the first and second divisions of the Hungarian national championship. Convenience sampling was used for data collection; hence, they were recruited personally (e.g., in Hungarian sport clubs) as well as using online forums (e.g., Facebook) and were asked to fill out the questionnaire. Before data collection, participants received information about the study, were assured of voluntary participation and anonymity, and provided online informed consent. The study was conducted in accordance with the Declaration of Helsinki and approved by the Institutional Ethics Committee of the Hungarian University of Sport Science (Ethical Approval Number: MTSE-OKE-KEB/01/2024).

### 2.2. Experimental Design

#### Questionnaires

The questionnaire on general menstrual cycle status was adapted based on the study of Jones et al. (2024) [1]. It included items on age at menarche, previous childbirth, cycle-related problems, the use of hormonal contraception and its reasons and menstrual cycle regularity. Specifically, participants were asked about menstrual pain occurring within 24–48 h after menstruation onset, pain within 7–10 days prior to menstruation, irregular menstruation, polycystic ovary syndrome (PCOS), endometriosis and adenomyosis.

Participants’ knowledge of the physiological aspects of the menstrual cycle, including typical cycle length and phases, was assessed using a questionnaire with a five-point Likert scale (1 = no tracking; 5 = usage of the symptothermal method and/or ovulation tests). Correct answers were also provided at the end of the questionnaire. In this section of the questionnaire, participants rated their agreement with five statements using a five-point Likert scale (1 = strongly disagree, 5 = strongly agree): “It is important for me to know which phase of the cycle I am in.”; “I feel shame when I have to talk or report about my menstruation or other parts of my menstrual cycle.”; “I would appreciate it if my team/coach considered my menstrual cycle when planning the training schedule.”; “I would appreciate it if my team/coach considered my menstrual cycle when evaluating my performance.”; and “My menstrual cycle has no effect on me at all”.

The impact of the menstrual cycle phases on physical and mental well-being was assessed using the Menstrual Distress Questionnaire (MEDI-Q) [14], supplemented with custom questions targeting (1) subjective performance: concentration difficulties, decreased training performance, increased training performance, feeling stronger, feeling weaker, feeling motivated, feeling explosive, impaired team performance, better team performance, avoiding digging/rolling, feeling slower, and feeling faster, and (2) injury susceptibility and capacity for recovery: feeling more vulnerable, better recovery, worse recovery. The MEDI-Q was administered for each cycle phase with explicit phase definitions provided before each set of questions as follows:

“How much do these statements apply to you DURING MENSTRUATION? Menstruation: days with bleeding (brownish spotting is not included). When responding, compared to the other phases of your menstrual cycle.

How much do these statements apply to you DURING FOLLICULAR PHASE? The follicular phase lasts from the end of menstruation to ovulation, which is approximately 10–14 days. When responding, compared to the other phases of your menstrual cycle.

How much do these statements apply to you DURING LUTEAL PHASE? The luteal phase lasts from ovulation to menstruation, approximately 12–16 days. When responding, compared to the other phases of your menstrual cycle.”

To capture the multidimensional nature of the training environment and subjective performance, we have constructed positive and negative training quality indices based on conceptually grouped questionnaire items. Specifically, items reflecting facilitative training perceptions (e.g., feeling stronger, faster, more explosive, improved team performance, faster recovery) were grouped as positive factors, whereas items reflecting impairments or constraints (e.g., feeling weaker, slower, impaired team performance, slower recovery, injury susceptibility, concentration difficulties, avoidance behaviors) were grouped as negative factors. For each respondent, the indices were calculated as the mean of the items within each category, thereby preserving the continuous nature of the data while reducing dimensionality. The factors incorporated in the index calculation and their distribution across categories are presented in Table 1.

### 2.3. Statistical Analysis

All statistical analyses were conducted using JASP statistical software (Version 0.17; JASP Team, Amsterdam, The Netherlands, 2023). Descriptive statistics were calculated, and non-parametric repeated-measures Friedman ANOVA was performed to assess within-participant differences across menstrual cycle phases, and a Bonferroni post hoc test was calculated to further define specific differences between datasets. Internal consistency and reliability of the composite indices were evaluated using Cronbach’s alpha. To provide further insight Kendall’s W values were also calculated for the Friedman ANOVA. The significance level was set at *p* < 0.05.

## 3. Results

Most of the participants (91.65%) stated that their menstrual cycle was regular. Seventy-five percent of participants reported at least one menstrual cycle-related problem: 59.5% experienced menstrual pain (within 24–48 h post-onset), 17.9% premenstrual pain, 15.5% irregular cycles (<21 or >35 days), 13.1% polycystic ovary syndrome (PCOS), 3.6% endometriosis, and 1.2% adenomyosis. The mean cycle length was 32.36 days (SD = 4.18), with a mean menstrual bleeding duration of 4.93 days (SD = 0.99). The longest cycle in the past 6 months had a mean of 33.42 days (SD = 8.42), while the shortest cycle had a mean of 25.13 days (SD = 5.37). In total, 10.7% used oral contraceptive pills, 1.2% hormonal vaginal rings, 20.2% had used oral contraceptives previously, and 67.9% had never used hormonal contraception. The main reasons for use were contraception (92.6%) and cycle regulation (25.9%); none reported use for performance optimization.

**Figure 1:**
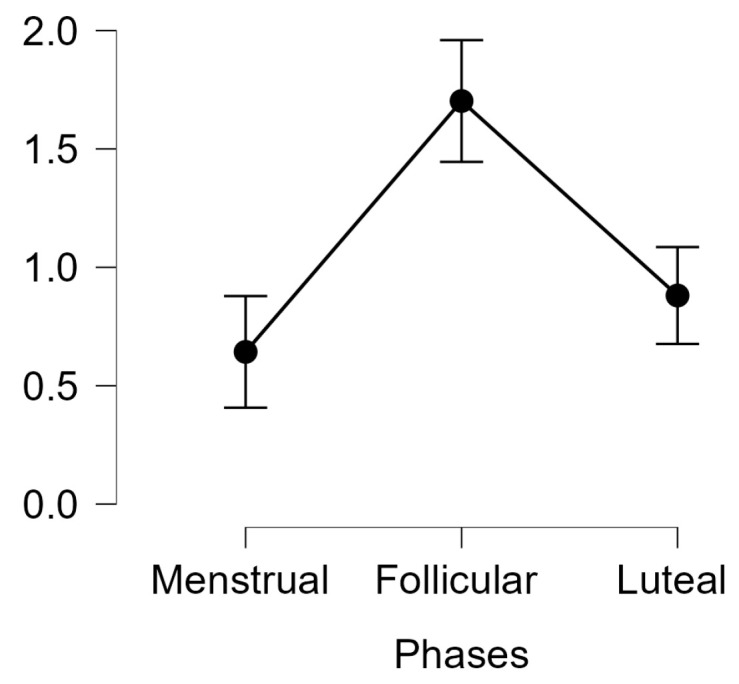
Subjective enhanced performance (mean ± SD). Significant difference was between Follicular–Menstrual and Follicular–Luteal while between Menstrual–Luteal the difference was not significant.

There were significant differences in the subjective performance results (*p* < 0.01, Kendall’s W = 0.211). Analyzing the subjective performance results, the highest subjective performance was measured during the follicular phase (M = 1.70, SD = 1.51), followed by the luteal phase (M = 0.88, SD = 1.13), and the lowest during menstruation (M = 0.64, SD = 1.00). Post hoc comparisons indicated that subjective performance was significantly higher in the follicular phase than in both the menstruation (*p* < 0.001) and luteal phases (*p* < 0.001), while the difference between menstruation and luteal phase was not significant (*p* = 0.329) (Figure 1).

**Figure 2:**
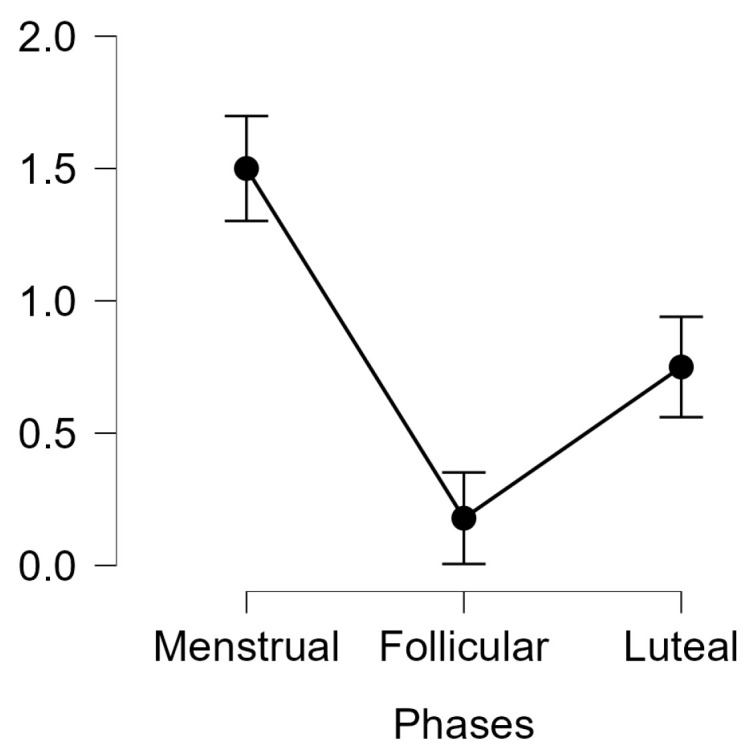
Subjective reduced performance (mean ± SD). Significant difference was observed between all comparisons.

Evaluation of subjective impaired performance resulted in significant phase differences (*p* < 0.01, Kendall’s W = 0.452), where the worst subjective performance was reported during the menstruation phase (M = 1.50, SD = 0.132), the least impaired/reduced subjective performance during the follicular phase (M = 0.18, SD = 0.47), and the intermediate luteal phase (M = 0.75, SD = 1.14). All post hoc comparisons were significant: reduced subjective performance in menstruation compared to both follicular and luteal phases (*p* < 0.001), and higher scores of impaired subjective performance in the luteal than in the follicular phase (*p* < 0.001) (Figure 2).

**Figure 3:**
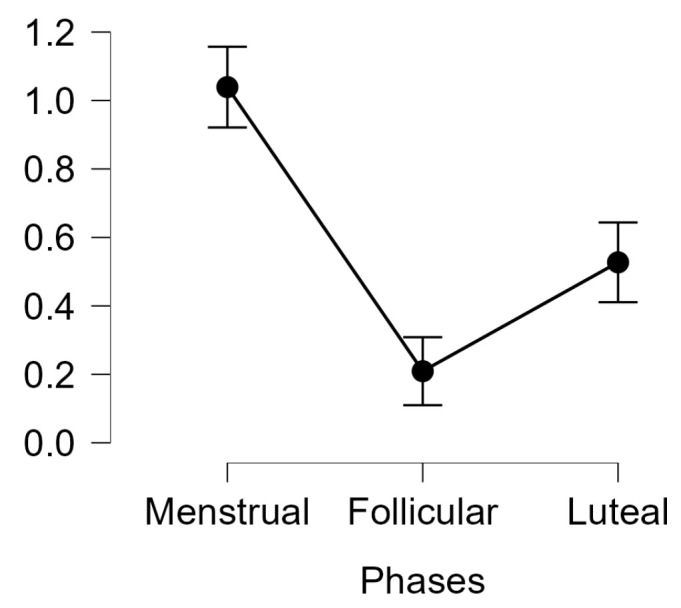
The negative training quality index across cycle phases (mean ± SD). Significant difference was observed between all comparisons.

Internal consistency of the composite indices indicated borderline but acceptable reliability for the positive training quality index (Cronbach’s α = 0.62) and acceptable reliability for the negative training quality index (Cronbach’s α = 0.70). There were significant differences in the negative training quality indexes (*p* < 0.01, Kendall’s W = 0.517). The negative training quality index was the worst during menstruation (M = 1.04, SD = 0.79), the best in the follicular phase (M = 0.21, SD = 0.36), and intermediate in the luteal phase (M = 0.53, SD = 0.81) (Figure 3). All between-phase comparisons were significant (all *p* < 0.001).

**Figure 4:**
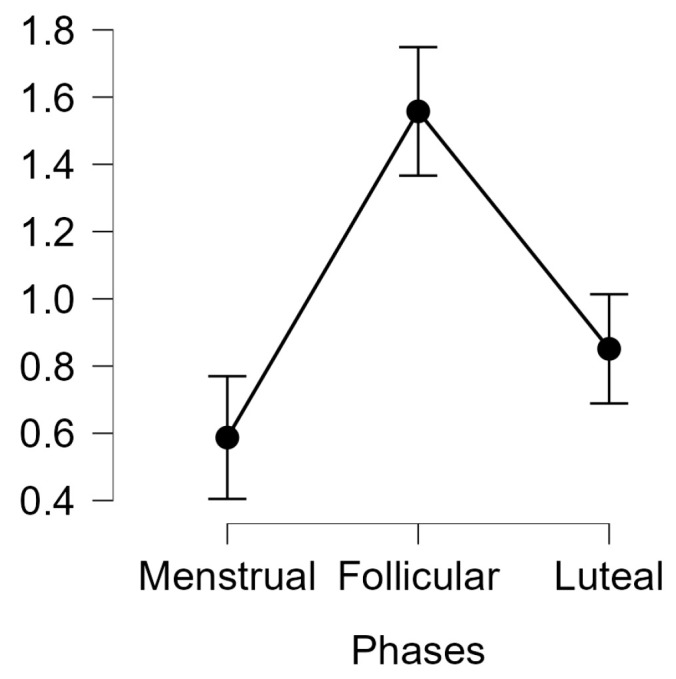
Positive training quality index across cycle phases (mean ± SD). Significant difference was between Follicular–Menstrual and Follicular–Luteal, while between Menstrual–Luteal the difference was not significant.

For the positive training quality index there were significant differences (*p* < 0.01, Kendall’s W = 0.184) and the highest values occurred in the follicular phase (M = 1.56, SD = 1.28), followed by the luteal (M = 0.85, SD = 1.03), and the lowest during menstruation (M = 0.59, SD = 0.69) (Figure 4). Post hoc tests showed the follicular phase was significantly higher than both menstruation (*p* < 0.001) and luteal (*p* < 0.001), but the luteal–menstruation difference was non-significant (*p* = 0.085).

**Figure 5:**
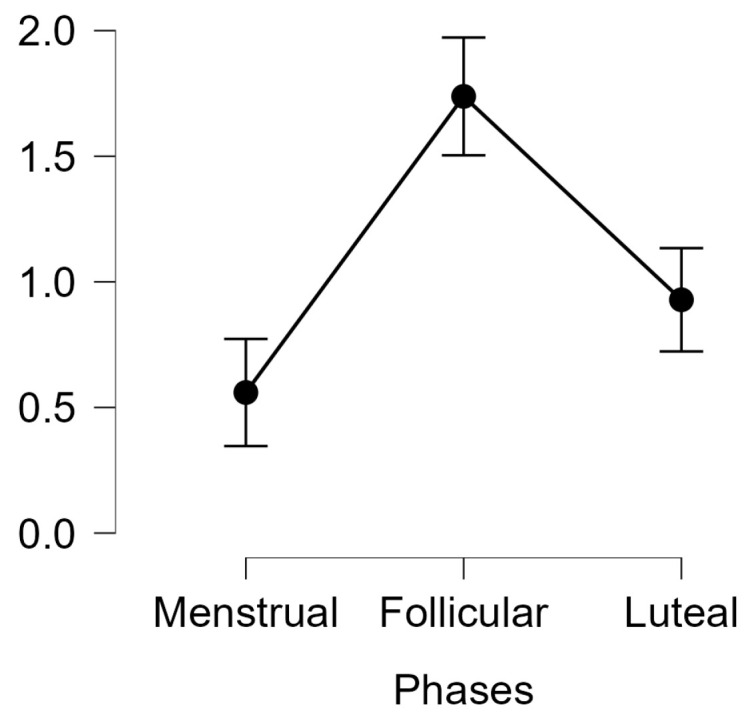
Motivation level across cycle phases (mean ± SD). Significant difference was between Follicular–Menstrual and Follicular–Luteal, while between Menstrual–Luteal the difference was not significant.

There were significant differences in the motivation levels (*p* < 0.01, Kendall’s W = 0.242). Motivation level was highest in the follicular phase (M = 1.74, SD = 1.55), moderate in the luteal phase (M = 0.93, SD = 1.20), and lowest during menstruation (M = 0.56, SD = 0.96) (Figure 5). A significant difference in motivation was observed between the menstruation and follicular phases (*p* < 0.001), and between the follicular and luteal phases (*p* < 0.001). However, there was no significant difference between the menstruation and luteal phases (*p* = 0.085).

We compared the responses obtained with the MEDI-Q questionnaire in our sample to the results reported by Cassioli (2023) [14]. In our data, mean symptom scores were consistently higher across all examined variables, with statistically significant differences in 17 symptoms (lower abdominal pain, breast tenderness, nausea, diarrhea, constipation, sadness, emotional lability, irritability/anger, impulsiveness, anxiety, increased appetite, decreased appetite, insomnia, hypersomnia, fatigue, decreased sexual drive, and concentration impairment). In contrast, three symptoms—muscle/osteoarticular pain (*p* = 0.11), headache (*p* = 0.36), and discomfort due to vaginal bleeding (*p* = 0.09)—did not differ significantly between the two studies.

**Figure 6:**
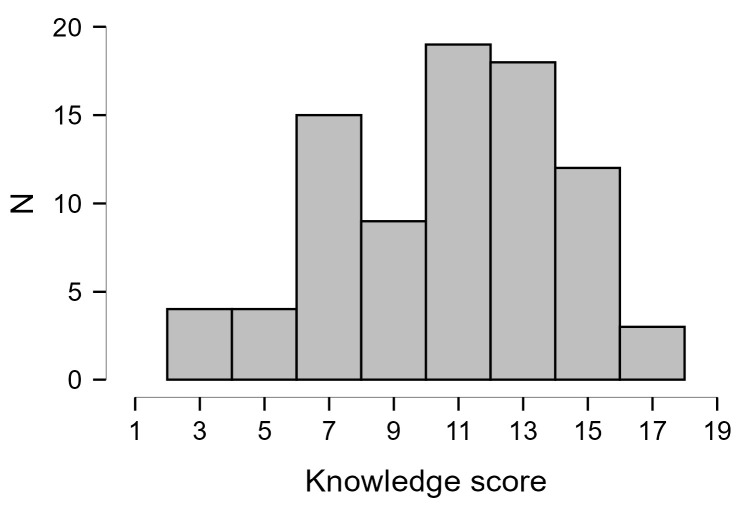
Distribution Cycle-related Knowledge Scores.

The mean education score considering knowledge about the menstruation cycle was 11.13 points (SD = 3.481). The median value was 12 points, indicating a slightly left-skewed distribution, as the mean was somewhat lower than the median. Respondents’ scores ranged from 3 to 18 points. Based on the visual representation of the distribution, the scores were mainly concentrated in the mid-range, with slight dispersion in both directions (Figure 6).

For the subjective perceptions and attitudes items, assessed on a 5-point Likert scale, participants reported the following mean scores: importance of knowing cycle phase (M = 2.60, SD = 1.24); embarrassment discussing menstruation (M = 0.75, SD = 1.17); desire for team/coach to consider cycle in training (M = 1.7, SD = 1.33); desire for team/coach to consider cycle in performance evaluation (M = 2.14, SD = 1.32); perceived lack of effect of cycle (M = 1.06, SD = 1.21) (Table 2).

## 4. Discussion

Results demonstrate significant fluctuations in multiple indices of perceived function and well-being across the menstrual, follicular, and luteal phases in volleyball players. Both enhanced and reduced sport performance indices varied significantly between cycle phases. The follicular phase was associated with the highest subjective sport performance and motivation, while the menstrual phase was associated with the lowest performance, the greatest reduction in sport ability, the most pronounced negative training quality, and the highest level of reported symptoms. The luteal phase consistently produced intermediate values. These patterns are consistent with previously described phase-related differences in hormonal milieu and symptom profiles [3,4,5]; however, given that we did not measure hormone concentrations, we interpret our results as subjective associations with menstrual cycle phase rather than direct evidence of underlying physiological mechanisms.

The negative training quality index was likewise highest during menstruation, while positive training quality and motivation were greatest during the follicular phase. Such findings corroborate the results from prior studies that identified increased perceived fatigue, pain, and emotional instability in the late luteal and menstrual phases [1,9,13,15]. Our results are not consistent with earlier findings, as they do not clearly indicate reduced performance in the early follicular phase; instead, they suggest that a personalized approach may be beneficial, with strategies adapted to each athlete’s individual response across the menstrual cycle [16]. Other studies indicate that both endurance and strength outcomes can vary across the cycle, yet results remain equivocal, some of them reporting better performance in mid-luteal or ovulatory phases, and others supporting a relative advantage during the follicular phase [15,17]. This inconsistency may stem from methodological heterogeneity, including differences in performance tests, phase classification, sample characteristics, and the extent to which individual symptom burden is accounted for. From a practical standpoint, recognizing menstruation and the late luteal phase as potential high-symptom periods, and the follicular phase as a relative window of perceived motivational and qualitative advantage, may support more individualized periodization strategies that respect athlete preference and symptom profiles rather than relying on a one-size-fits-all menstrual cycle model [16,18,19].

Prevalence rates for menstrual cycle problems—including dysmenorrhea, premenstrual pain, cycle irregularity, PCOS, endometriosis, and adenomyosis—were high in the present sporting cohort. The most frequently reported menstrual-related issue was menstrual pain within 24–48 h following menstruation onset (59.52%), followed by pain 7–10 days before menstruation (17.86%) and irregular menstruation with cycle length shorter than 21 days or longer than 35 days (15.48%). Polycystic ovary syndrome (PCOS) was reported by 13.10% of participants, while endometriosis and adenomyosis affected 3.57% and 1.19%, respectively. While 25.00% of the participants reported none of these conditions. This prevalence is higher than previously reported by De Souza et al. (2010) [6], who found that about half of exercising women experienced subtle or severe menstrual disturbances. One possible explanation is that our athletes were more body-conscious and paid closer attention to changes. Another reason may be that they felt more comfortable reporting symptoms, whereas menstrual problems are often underreported by athletes. Our broader definition of “menstrual cycle problems,” which included clinically diagnosed conditions such as PCOS and endometriosis, may also have contributed to the higher prevalence.

The present sample reported higher average symptom burdens during menstruation than previously described in the reference MEDI-Q data [14], reinforcing that the impacts of the menstrual phase remain a salient barrier to performance and general well-being for many athletes, even in highly competitive contexts. These results support targeted symptom management and proactive phase-specific support as important components of female athlete care.

Participants rated knowledge of menstrual cycle phases as moderately important (M = 2.60, SD = 1.24), with substantial variability indicating that some athletes view cycle awareness as essential for training and performance, whereas others regard it as less relevant to their sport, echoing the heterogeneous attitudes reported by Findlay et al. (2020) [20]. Most players reported feeling relatively comfortable discussing menstruation (M = 0.75, SD = 1.17), yet the large standard deviation suggests that a subgroup still experiences embarrassment or reluctance, consistent with recent evidence that menstrual health remains a sensitive topic in many sport environments and that communication is often hindered by limited knowledge and confidence among both athletes and coaches [12,21,22,23]. Athletes expressed a moderate desire for coaches or teams to consider their cycle in training (M = 1.79, SD = 1.33), but a stronger preference for cycle-related context in performance evaluation (M = 2.14, SD = 1.32), indicating that they prioritize fair, cycle-informed appraisal over extensive training adaptations. This pattern suggests a pragmatic entry point for practice: integrating menstrual cycle information into monitoring and performance debriefs as a first step, before moving toward more individualized training adjustments where appropriate, in line with current recommendations on menstrual health literacy and cycle tracking in sport. Despite reporting relatively low perceived effects of the cycle on their performance (M = 1.06, SD = 1.21), these findings likely reflect heterogeneous symptom experiences and variable awareness rather than the absence of meaningful effects, reinforcing calls for targeted educational initiatives and structured communication strategies to help athletes and coaches to better recognize, discuss, and, where needed, accommodate menstrual-related symptoms in training and competition [24,25,26].

From a sport-specific perspective, these findings are particularly relevant for volleyball, where athletes must train and compete across all menstrual cycle phases without the possibility of strategically avoiding high-symptom periods. In this context, the observed phase-related differences in perceived performance, training quality, and symptom burden highlight concrete opportunities for coaches to adjust practice loads, match preparation, and recovery strategies in ways that better accommodate the demands of competitive volleyball. By focusing on a homogeneous sample of mid- and high-level volleyball players, the present study extends previous work conducted in mixed-sport or non-athlete samples and provides sport-specific insights that may be more directly translatable to volleyball performance environments.

## 5. Limitations and Future Directions

While this study benefits from a well-characterized cohort and repeated measures design, it is limited by self-report biases, selection bias introduced by convenience sampling, the lack of objective performance measures, and the possible influence of sport-specific routines. Furthermore, the lack of valid menstrual phase classification, such as hormonal verification or a validated tracking method, introduces misclassification bias. This study did not assess sleep quality, sleep quantity, or stress levels, which may have affected the results. Importantly, as attitudes regarding menstrual cycle integration in sports remain divided, future interventions should respect individual preferences while promoting informed, evidence-based practice. We must also mention that as the used MEDI-Q questionnaire is a modified version, we do not possess the appropriate Cronbach Alpha values for determining reliability. Biological assessment (e.g., hormone confirmation of phases), larger samples, and additional objective outputs are warranted for future research. The guidance on menstrual suppression explicitly notes that combined hormonal contraceptives might be used to control bleeding for conditions such as abnormal uterine bleeding, endometriosis, or irregular cycles, highlighting their capacity to mask underlying menstrual dysfunction. Because the aim of our study was to identify the effect of menstrual cycle phases on perceived performance we decided not to discard those limited number of participants who are regular users of HC.

## 6. Conclusions

This cross-sectional study suggests that female volleyball athletes report meaningful menstrual phase-related differences in subjective performance, training experiences, and symptom burden. These exploratory findings, based solely on self-reported perceptions, indicate that menstrual cycle phase may be a relevant consideration when reflecting on training experiences and performance appraisal, rather than providing evidence for causal effects. Based on this, phase-aware education and individually tailored, athlete-led discussions may be useful tools for coaches and support staff who wish to better align training and performance evaluation with athletes’ perceived experiences, while recognizing the need for future research using objective measures and longitudinal designs.

## Figures and Tables

**Table 1:** Factors of Negative and Positive Training Quality.

Factors of Negative Training Quality	Factors of Positive Training Quality
Feeling weaker	Feeling stronger
Impaired team performance	Better Team Performance
Avoid digging/rolling	Feeling explosive
Feeling slower	Feeling faster
Slower recovery time	Faster Recovery Time
Injury susceptibility	
Concentration difficulties	

**Table 2:** Results for subjective perception and attitude items.

Items	Mean	SD
Importance of knowing cycle phase	2.6	1.24
Embarrassment discussing menstruation	0.75	1.17
Desire for team/coach to consider cycle in training	1.79	1.33
Perceived lack of effect of cycle	1.06	1.21

## Data Availability

The data presented in this study are available on request from the corresponding author due to information that could compromise the privacy of research participants.
